# Strong scale‐dependent relationships between fine‐root function and soil properties uncovered with spatially coupled sampling

**DOI:** 10.1111/nph.70143

**Published:** 2025-04-29

**Authors:** Caroline Dallstream, Lola Milder, Jennifer S. Powers, Fiona M. Soper

**Affiliations:** ^1^ Department of Biology McGill University Montreal QC H3A 1B1 Canada; ^2^ Bieler School of Environment McGill University Montreal QC H3A 2A7 Canada; ^3^ Department of Plant and Microbial Biology University of Minnesota Saint Paul MN 55108 USA

**Keywords:** arbuscular mycorrhiza, magnesium, phosphatase, plant–soil interactions, root physiology, root traits, tree, tropics

## Abstract

- Substantial fine‐root trait variation is found at fine spatial scales but rarely linked to edaphic variation. We assessed the spatial scales of variation in fine‐root traits and adjacent soils using a spatially coupled, nested sampling scheme along a fertility gradient in a seasonally dry tropical forest tree, *Handroanthus ochraceus*. We examined relationships among fine‐root traits and identified edaphic drivers of fine‐root function.
- We collected fine‐root samples at three scales: multiple samples within individual trees (separated by > 1 m), among trees in a site (3–60 m) and across three sites (15–60 km). We quantified physiological, symbiotic, morphological, chemical and architectural traits, and paired soil physical and chemical properties.
- Fine‐root traits and soils often varied most at fine spatial scales. Root arbuscular mycorrhizal colonization and phosphomonoesterase activity were coordinated and driven by coarse‐scale heterogeneity in bulk density, magnesium and phosphate. The trade‐off between large diameter and high specific root length, respiration rate and nitrogen concentration was driven by fine‐scale heterogeneity in ammonium.
- The role of base cations was notable, with nitrogen and phosphorus being less influential than expected. Intraspecific fine‐root responses to edaphic properties can occur at multiple spatial scales simultaneously and be detected when variation in both is properly captured and spatially matched.

## Resumen

- Se observa una variación sustancial en los rasgos de las raíces finas a escalas espaciales reducidas, aunque rara vez esta se asocia con propiedades edáficas. Evaluamos las escalas espaciales de variación en los rasgos de las raíces finas y en los suelos adyacentes mediante un diseño de muestreo anidado y espacialmente acoplado, a lo largo de un gradiente de fertilidad en un árbol de bosques tropicales estacionalmente secos, *Handroanthus ochraceus*. Analizamos las relaciones entre los rasgos de las raíces finas e identificamos los factores edáficos que impulsan su funcionamiento.
- Se recolectaron muestras de raíces finas en tres escalas: múltiples muestras dentro de árboles individuales (separadas por >1 m), entre árboles dentro de un mismo sitio (3–60 m) y entre tres sitios diferentes (15–60 km). Se cuantificaron rasgos fisiológicos, simbióticos, morfológicos, químicos y arquitectónicos, junto con propiedades físicas y químicas del suelo emparejadas.
- Los rasgos de las raíces finas y las propiedades del suelo variaron con mayor frecuencia a escalas espaciales finas. La colonización micorrícica arbuscular de las raíces y la actividad fosfomonoesterasa estuvieron coordinadas y fueron impulsadas por la heterogeneidad a gran escala de la densidad aparente, el magnesio y el fosfato. A escala fina, el intercambio entre un mayor diámetro radicular y una elevada longitud específica de raíz, junto con la tasa de respiración y la concentración de nitrógeno, estuvo influenciado por la heterogeneidad del amonio.
- El papel de los cationes base fue destacado, mientras que el nitrógeno y el fósforo resultaron ser menos influyentes de lo esperado. Las respuestas intraespecíficas de las raíces finas a las propiedades edáficas pueden ocurrir simultáneamente en múltiples escalas espaciales, y ser detectadas cuando la variación en ambas dimensiones se captura adecuadamente y se empareja espacialmente.

## Introduction

**Fig. 1:**
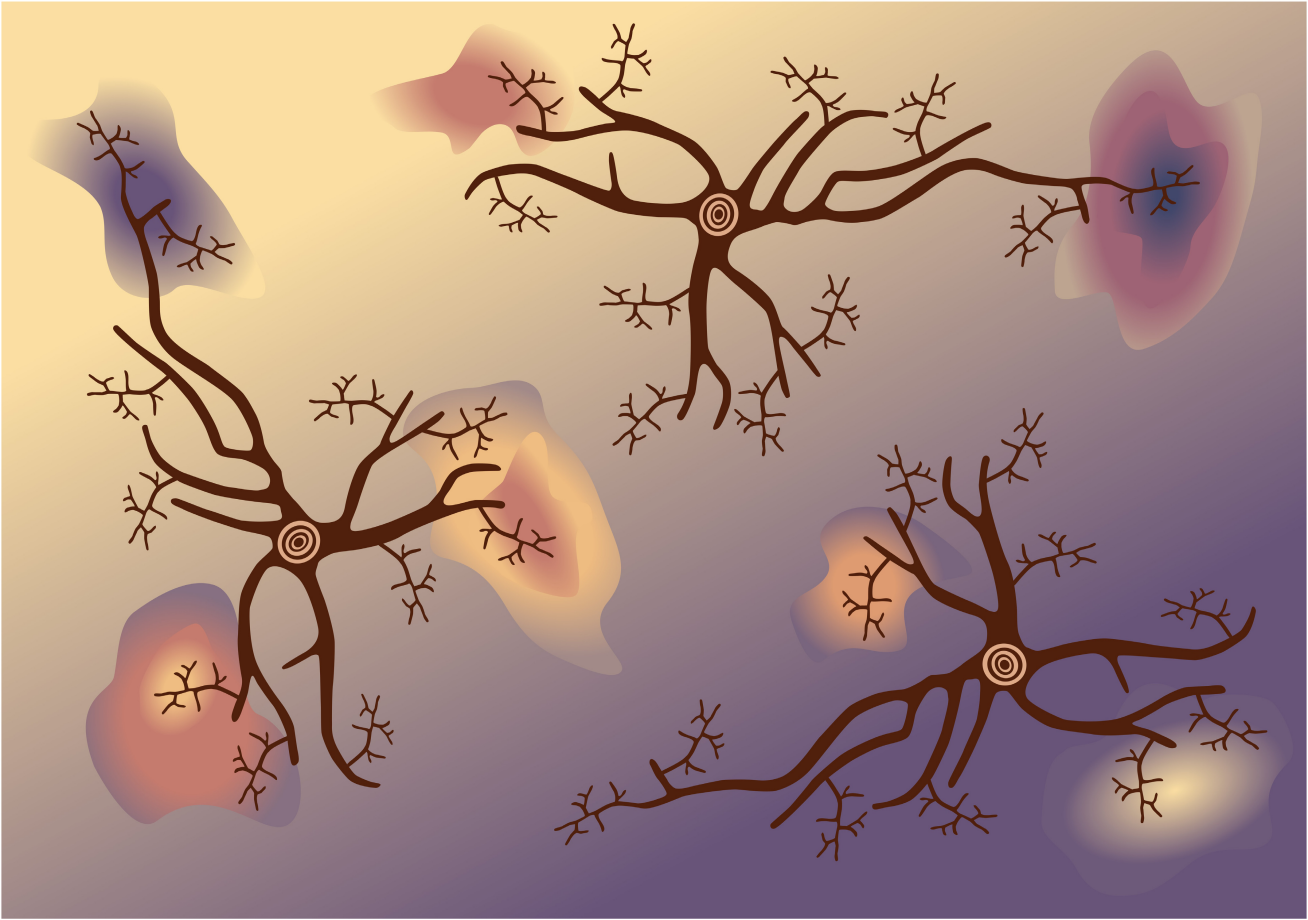
Conceptual representation of heterogeneity in a soil property that extensive root systems could encounter. Soil properties can be relatively homogeneous, or heterogeneity can be patterned as gradients, patches or combinations of both (shown here) at various spatial scales. Colors represent the relative values of a single soil resource or physical characteristic from low (light) to high (dark).

Trait‐based ecology often seeks to define organisms' responses to broad gradients, assuming that coarse‐scale biotic and abiotic conditions are the dominant drivers of their distribution and functioning (Shipley *et al*., 2016). Fine‐root trait research has historically operated under this assumption that coarse gradients drive function, leading to a frequent spatial mismatch between fine‐root and environmental sampling. However, this practice may substantially hinder the identification of certain influences on fine roots, the smallest and most distal modular units of the root system (by one definition < 2 mm diameter (D); Freschet *et al*., 2021). Fine roots must balance multiple functions: localizing and acquiring water and all essential plant nutrients, competing with microbes and other plants, defending against pathogens and interfacing with belowground symbionts. This multifunctionality is overlaid on a highly heterogeneous soil environment. A single edaphic variable, whether it is a resource or physical characteristic, could be relatively homogeneously or heterogeneously distributed,  and this heterogeneity can also occur in various patterns – gradients, patches or combinations of both – that manifest at various spatial scales (Fig. 1; Ettema & Wardle, 2002; Yavitt *et al*., 2009). Given the small size and modular nature of fine roots, and the immense heterogeneity of the soil environment they navigate, it is highly probable that fine roots plastically adjust their functioning in response to the soil environment they directly encounter (de Kroon *et al*., 2005). Aligning the spatial scales of fine‐root and soil samples could thus expose the clearest fine‐root functional responses to edaphic drivers, particularly those that occur at fine spatial scales.

Individual edaphic variables often have independent degrees and spatial patterns of heterogeneity, forming a mosaic across the landscape (Fig. 1; Ettema & Wardle, 2002; Yavitt *et al*., 2009). Overlaid, multiple variables thus form a multidimensional soil mosaic. For soil nutrients, the source of inputs to ecosystems is likely to structure their spatial distributions. Beyond the spatial structuring that may result from the source, distributions could be restructured by the effects of microtopography, plant nutrient cycling, decomposition or animal activity (Roy & Singh, 1994; Waring *et al*., 2015; Osborne *et al*., 2017). Phosphorus (P) and exchangeable base cations, including magnesium (Mg), potassium (K) and calcium (Ca), primarily enter ecosystems from the weathering of parent materials. The type and age of parent materials and the climatic conditions that influence weathering rates thus tend to drive the quantities and patterns of these resources (Walker & Syers, 1976; Kaspari & Powers, 2016; Waring *et al*., 2021). For example, as soils age, total P and plant‐available inorganic orthophosphate (PO_4_) decline as rocks are heavily weathered and P is transformed into less‐accessible forms or leached (Walker & Syers, 1976). As soils age, total nitrogen (N) increases (Walker & Syers, 1976). Nitrogen can also be weathered from parent materials, but the primary natural input into ecosystems is biological N fixation, which tends to structure heterogeneity at finer spatial scales resulting from plant activity and litter and microbial ‘hotspots’ of activity (Vitousek *et al*., 2013; Waring *et al*., 2015, 2016, 2021; Akana *et al*., 2023).

Due to the broad relationships between ecosystem age and total N and P, the tropics are expected to be primarily limited by P (Vitousek *et al*., 2010; Dalling *et al*., 2016; Cunha *et al*., 2022). However, readily available plant inorganic N – extractable nitrate (NO_3_) and ammonium (NH_4_) – could also shape plant responses; N can be highly variable in space and has been shown to influence tropical plant fine‐root biomass, morphology and arbuscular mycorrhizal colonization (Wurzburger & Wright, 2015; Waring *et al*., 2016). In a broader sense, due to their multifunctionality, it is probable that fine roots respond to multiple soil variables at once. This is perhaps especially likely in the tropics, where nutrient colimitation may be more common due to the long‐term weathering of all rock‐derived nutrients (Kaspari & Powers, 2016).

Despite the complexity of the soil environment, fine‐root studies have tended to sample roots at finer spatial resolutions than soils. For example, a study along a 1000‐m elevational gradient in the French Alps sampled fine roots in five 400 m^2^ plots per elevation separated by 300–2000 m of distance, but soil cores collected from plots were pooled to each elevation; this spatial mismatch could have obscured or blurred some responses, especially considering that most intraspecific fine‐root trait variation was seen within elevations rather than among them (Weemstra *et al*., 2021). Another study in British Columbia, Canada, found the majority of intraspecific fine‐root trait variation occurred within individual trees, rather than across a *c*. 220‐km latitudinal gradient (Defrenne *et al*., 2019). It is thus clear that fine roots can vary significantly at fine spatial scales, but the causes of this variation are not.

There is also evidence that certain soil properties can vary the most at fine spatial scales, while others predominantly vary at coarse ones. A N cycle study conducted in seasonally dry neotropical forests found ≥ 50% of variation for 13 of the 16 soil variables occurred within 300 m^2^ subplots rather than among sites separated by 60 km, even though sites had functionally distinct plant communities (Waring *et al*., 2016). In a temperate forest, soil NO_3_ and NH_4_ varied substantially at spatial scales < 1 m, considerably smaller than the root system of juvenile trees (Akana *et al*., 2023). These trends of high soil heterogeneity at fine spatial scales coupled with high fine‐root trait variation at fine biological (i.e. within individuals or within species) or spatial scales could be directly related. Hence, to accurately and consistently describe fine‐root functional responses to edaphic gradients, it is likely that soil sampling resolution needs to closely match fine‐root sampling resolution.

In addition to being multifunctional, fine‐root traits are also multidimensional (Kramer‐Walter *et al*., 2016; Bergmann *et al*., 2020; Carmona *et al*., 2021; Weigelt *et al*., 2021). Across species globally, some traits have been shown to express multiple trade‐offs and coordinations, such that high specific root length (SRL) trades off with large D and high arbuscular mycorrhizal colonization intensity (AM%), and independently, high root tissue density (RTD) trades off with high N concentration (N%); these trade‐offs are interpreted as a do‐it‐yourself vs outsourcing trade‐off with respect to symbiotic dependence, and a conservation trade‐off between fine roots with high longevity vs high metabolic activity (Bergmann *et al*., 2020). This multidimensionality appears to stem from fine roots' cylindrical shape, cortex allometry and the flexibility of cell wall thickness and number, which may permit immense trait diversity and plasticity (Zhang *et al*., 2024). Since these intrinsic relationships among fine‐root traits could constrain their responses to the environment, they should be explicitly considered in research design.

Besides constraining potential fine‐root responses, strong relationships among fine‐root traits could allow for measurement proxies for harder‐to‐measure traits and be leveraged to statistically impute data for less commonly measured fine‐root traits based on a wealth of existing morphological data (Iversen *et al*., 2021). Although physiological fine‐root traits like root respiration rate (RESP) and potential acid phosphomonoesterase activity rate (PME) capture direct plant impacts on biogeochemical cycles of carbon, N and P, and directly reflect plant energetic costs and potential nutrient acquisition, measuring them in the field is complicated and time sensitive. As a result, fine‐root data are biased toward traits such as D, SRL, RTD and N%. Measuring suites of fine‐root traits on each fine‐root sample could help to identify common traits as proxies for physiological, symbiotic or other traits more directly related to functioning.

Prior works have sought to understand fine‐root trait trade‐offs and coordinations in isolation to explain variation generally, but a more specific approach is to relate fine‐root variation to environmental conditions. Recent works are striving to relate individual traits and the multidimensional fine‐root trait space to edaphic variation to advance theory by contextualizing these trade‐offs ecologically (Laliberté, 2017; Guilbeault‐Mayers & Laliberté, 2024). Fine‐root responses to edaphic variables are often complex, with some evidence that acquisition is downregulated as edaphic resources increase and other evidence showing the opposite. In fact, extremely nutrient‐limited ecosystems can host the greatest diversity of nutrient‐acquisition strategies (Zemunik *et al*., 2015; Dallstream *et al*., 2023). For example, PME has been found to be downregulated with increasing soil P or P addition in tropical forests, indicating acquisitiveness was increased under limitation (Ushio *et al*., 2015; Guilbeault‐Mayers *et al*., 2020; Cabugao *et al*., 2021; Lugli *et al*., 2021). However, along a soil chronosequence in New Zealand, SRL was similar between high‐ and low‐P sites, but in the low‐P sites, RTD increased and root N and P decreased, indicating that some acquisitiveness was decreased under limitation (Holdaway *et al*., 2011). For this reason, defining relationships between individual fine‐root traits and edaphic properties is important, but the interpretation of plant acquisitiveness requires consideration of many traits in concert.

The main goal of the present study was to identify the dominant spatial scales of variation for fine‐root traits and soil variables. A secondary goal was to identify intrinsic drivers of fine‐root traits (i.e. fine‐root trait coordinations and trade‐offs) including fine‐root trait proxies for physiological functions and other root–root relationships. Finally, we aimed to identify the edaphic drivers of fine‐root function (i.e. root–soil relationships) and to examine how spatial scales of variation affected these relationships. Such questions about the intrinsic and edaphic drivers of fine‐root trait variation are especially relevant for tropical forests, which disproportionately contribute to global carbon sequestration and will regulate feedbacks to climate change (Wieder *et al*., 2015; Allen *et al*., 2020). The tropics are also considered unique in terms of the abiotic factors and mineral nutrients expected to limit plant productivity, but are relatively under‐sampled and underrepresented in global and vegetation models (Cusack *et al*., 2024).

We employed a nested, high‐resolution sampling scheme that coupled fine‐root samples to their adjacent soils across an edaphic gradient in the seasonally dry tropical forests of northwestern Costa Rica. Sampling scale spanned from within trees (> 1 m), to among trees within a site (2.5–60 m), to among sites (15–60 km). We sampled a single tree species, *Handroanthus ochraceus*, that is common and widely distributed in the region and thus perhaps more likely to express substantial trait variation (Powers *et al*., 2009). To parsimoniously evaluate root–root and root–soil relationships, we emphasized multivariate analyses given the multidimensional nature of both. Multiple P acquisition mechanisms were quantified due to their putative importance in shaping tropical fine‐root responses: SRL and AM% increase the volume of soil accessed for a given carbon construction cost, which helps plants acquire low‐mobility nutrients, such as PO_4_, whereas PME mineralizes PO_4_ from organic substrates (i.e. phosphomonoesters).

We hypothesized the following. (1) Substantial fine‐root trait variation would occur within the finest of the three sampling scales (i.e. greater than one‐third of total variance within trees), and substantial soil variation would occur at the finest of two sampling scales (i.e. greater than one‐half of total variance within sites). (2) As observed for interspecific trait relationships, intraspecific fine‐root trait relationships would also show that D and AM% coordinate with each other and trade off with SRL and PME. Independently, RTD would trade off with N% and RESP. (3) Fine‐root traits related to P acquisition would be downregulated as soil PO_4_ increased. For example, we expected that PME, AM% and/or SRL would decrease as plant‐available soil PO_4_ increased. Generally, root morphological traits would become more conservative as soil PO_4_, NH_4_ and NO_3_ increased; for example, SRL would decrease and RTD would increase. (4) If the spatial scales of soil and fine‐root trait variation are similar and properly captured, the root–soil relationships observed should be stronger.

## Materials and Methods

### Study sites

Field sampling was conducted in the early rainy season in seasonally dry tropical forests in the Guanacaste province of Costa Rica in June and July 2022. The three study sites have a mean annual temperature of 25°C with wet seasons from ~ May–November with high interannual variability in precipitation (Gillespie *et al*., 2000; Powers *et al*., 2009; Waring *et al*., 2019). Sampling was performed in three sites along a fertility gradient with similar annual precipitation: Santa Rosa National Park (10.84°N, 85.62°W, mean annual precipitation (MAP) 1575 mm), Horizontes Experimental Forest Station (10.71°N, 85.57°W, MAP 1800 mm) and Palo Verde National Park (10.38°N, 85.32°W, MAP 1490 mm; Gillespie *et al*., 2000; Powers *et al*., 2009; Waring *et al*., 2019). Sites were in the Área de Conservación Guanacaste or the Área de Conservación Arenal Tempisque. Soils in the three sites are mainly volcanic Inceptisols and Vertisols (Powers *et al*., 2009).

*Handroanthus ochraceus* (Cham.) Mattos (Bignoniaceae) is a widely distributed neotropical tree that is common in dry and seasonally dry forests (Gentry, 1992; Madeira *et al*., 2009; Powers *et al*., 2009). *H. ochraceus* is deciduous and can grow 4–12 m tall (Gentry, 1992). It was one of the five most common species in forests < 30 yr old in the Palo Verde site and, in Brazil, is common across early to late successional stages (Madeira *et al*., 2009; Powers *et al*., 2009).

### Fine‐root sampling and trait analyses

**Fig. 2:**
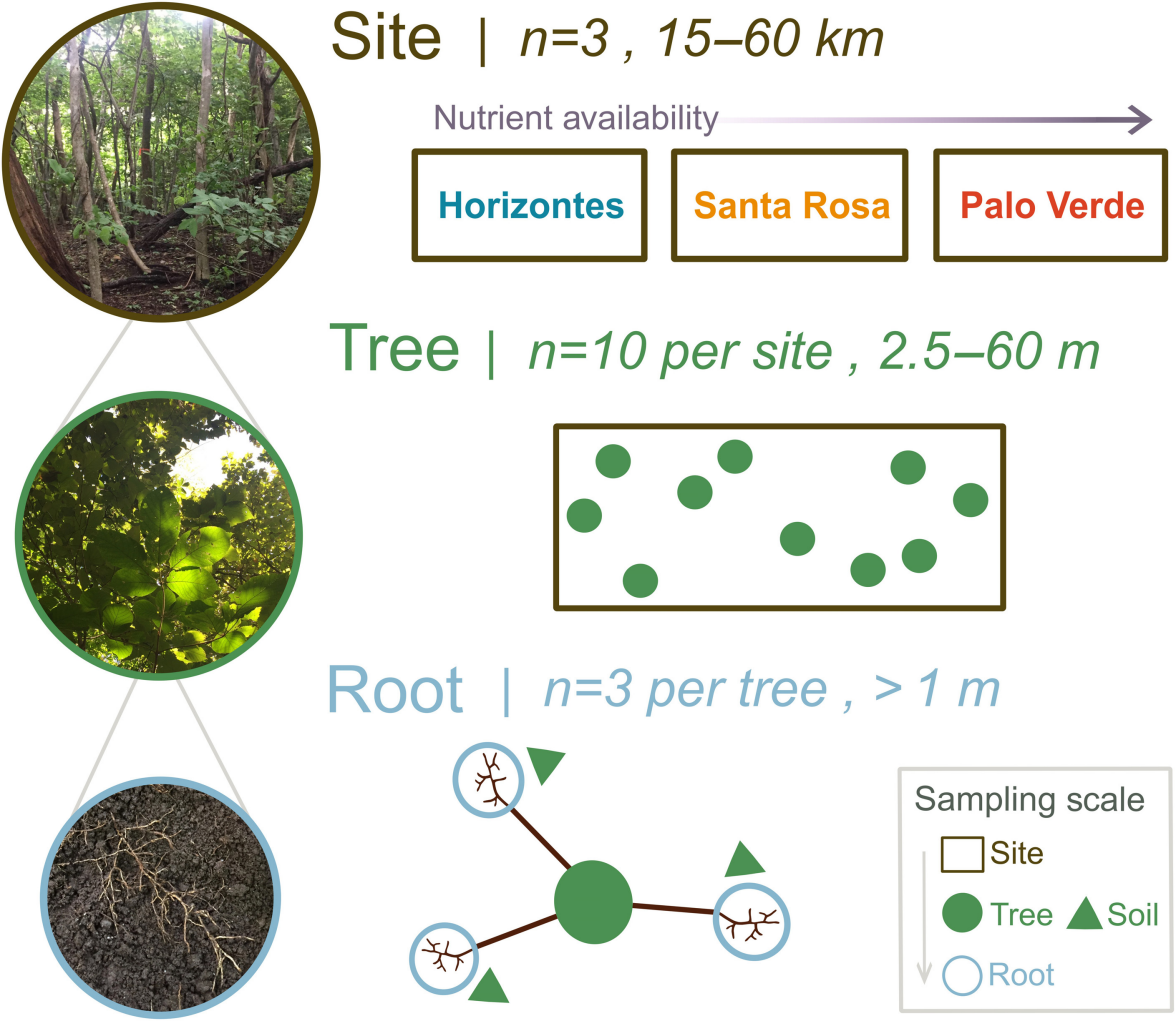
Representation of the nested sampling scheme employed: the three sites were distributed along a gradient of nutrient availability shown by principal component analysis of soil variables (Fig. 5a), and 10 trees of *Handroanthus ochraceus* were sampled per site, with three fine‐root samples per tree (*n* = 30 trees, *n* = 90 fine roots). Fine‐root sample sizes are indicated, as are the distances between sites, trees and fine roots. The color of text corresponds to fine‐root and soil sampling scales (i.e. soils were collected adjacent to fine‐root samples but pooled to the tree level for analyses; *n* = 30 soil samples).

In each of the three sites, we traced the roots of 10 *H. ochraceus* trees with diameter at breast height (DBH) between 5.5 and 17.7 cm to obtain three fine‐root samples from the upper 10 cm of mineral soil that were separated by at least 1 m (Fig. 2; Table 1; *n* = 90). Fine roots were considered as the first three root orders (i.e. nonwoody absorptive roots) following the morphometric classification system. Distance between sites ranged from 15 to 60 km. Site areas were 2295 m^2^ (Horizontes), 2240 m^2^ (Santa Rosa) and 1275 m^2^ (Palo Verde). Average distance between trees within a site ranged from 2.5 to 60 m. Mean tree DBH ± SD (cm) for the sites was 11.0 ± 1.9 (Horizontes), 10.5 ± 4.2 (Santa Rosa) and 10.7 ± 2.6 (Palo Verde).

**Table 1:** Soil variables and fine‐root traits considered in this study, their abbreviations, sample sizes (N) and units.

Variable or trait	Abbreviation	*N*	Unit
Soil Hedley P fractions	–	15	mg kg^−1^
Soil texture fractions	–	15	%
Soil bulk density	Bulk	30	g cm^−3^
Soil exchangeable calcium cations	Ca	30	cmol kg^−1^
Soil gravimetric water content	GWC	30	g g^−1^
Soil exchangeable potassium cations	K	30	cmol kg^−1^
Soil exchangeable magnesium cations	Mg	30	cmol kg^−1^
Soil total N concentration	N%	30	%
Soil extractable ammonium‐N	NH_4_	30	mg kg^−1^
Soil extractable nitrate‐N	NO_3_	30	mg kg^−1^
Soil pH in water	pH	30	–
Soil extractable orthophosphate‐P	PO_4_	30	mg kg^−1^
Root arbuscular mycorrhizal colonization	AM%	90	%
Root diameter	D	90	mm
Root nitrogen concentration	N%	90	%
Root potential acid phosphomonoesterase activity rate	PME	90	μmol pNP g^−1^ h^−1^
Root branching intensity	RBI	90	tips cm^−1^
Root respiration rate	RESP	90	nmol CO_2_ g^−1^ s^−1^
Root tissue density	RTD	90	g cm^−3^
Specific root length	SRL	90	m g^−1^

Rows are arranged by sample sizes and then by abbreviations alphabetically.

Fine‐root tissue was divided into two subsamples for trait analyses. Within 5 min after harvesting, a *c*. 1 g subsample was washed in tap water and gently blotted dry to measure RESP with a LI‐6800 (Li‐Cor; Lincoln, NE, USA) in the insect respiration chamber attachment (6800‐89). Chamber conditions were set to 30°C to approximate ambient air temperature and 50% relative humidity, and samples did not show any signs of desiccation during the short measurement duration (*c*. 15 min). This subsample was dried at 60°C for 72 h and weighed to standardize rates. Later, the subsample was rehydrated in tap water overnight and scanned on a flatbed scanner (V850; Epson, Nagano, Japan) at 600 DPI (Guilbeault‐Mayers *et al*., 2020). To quantify morphological traits, scans were analyzed with rhizovision explorer v.2.0.3 (Seethepalli *et al*., 2021). Then, the subsamples were stained for mycorrhizal quantification via the ink and vinegar method: Roots were cleared in 10% KOH in a 90°C water bath for *c*. 15 h, rinsed, acidified in 1% acetic acid and stained in 5% Sheaffer ink solution for 7 min each, destained in 50% lactoglycerol and finally mounted in glycerin jelly (Widden, 2001). Arbuscular mycorrhizal colonization intensity was quantified as a percentage of the presence of mycorrhizal structures in 100 fields of view at 200× magnification (McGonigle *et al*., 1990).

A second *c*. 1 g fine‐root subsample was immediately placed into 50 mM CaSO_4_ solution and stored in a cooler in the field, and then refrigerated (Nasto *et al*., 2017; Soper *et al*., 2019). Potential acid phosphomonoesterase activity rates were determined using the *para*‐nitrophenyl phosphate (pNPP) protocol within 3 d of harvesting (*sensu* Cabugao *et al*., 2021). Fine roots were gently washed in tap water and split into two 0.5 ± 0.1 g analytical subsamples and placed into 50 mM acetic acid buffer (pH 5.0). Samples received 50 mM pNPP substrate. Controls for root, buffer and pNPP color were included. Fine roots were shaken for 2 h at 28°C, and reactions were terminated with 0.11 M NaOH. Absorbances were measured with a field spectrophotometer at 405 nm and compared with the colorimetric product, *para*‐nitrophenol (pNP; Walter Products Inc., Plymouth, MI, USA). Analytical subsamples were dried at 60°C for 72 h and weighed, and then recombined and ground in a ball mill to determine carbon and N concentration with an elemental analyzer at the Cornell University Stable Isotope Laboratory (Carlo Erba, Milan, Italy).

### Soil variable analyses

Two sets of soil cores from the upper 10 cm of mineral soil were collected adjacent to each of the three fine‐root samples per tree (i.e. soils were sampled from undisturbed soils within 5 cm of the edge of fine‐root excavations). Each set of three cores was pooled to the tree level (*n* = 30). Soil core pooling was performed because we hypothesized that fine roots would respond more strongly to P than N (and we expected P to vary at coarser spatial scales than N), and due to logistical constraints imposed by time‐sensitive measurement of physiological fine‐root traits and soil extraction. A 2‐cm diameter core set was collected to quantify bulk density and gravimetric water content (GWC). A 5‐cm diameter core set was collected for nutrient, Hedley P fractionation, pH and texture analyses. Most soil analyses had 30 samples, excluding soil texture and Hedley P fractionation for which there were 15 samples because the two closest trees were pooled within every site (Table 1).

To determine bulk density and GWC, the 2‐cm set of cores was pooled to the tree level and homogenized and sieved through a 2‐mm mesh sieve. What did not pass through the mesh was considered stone content, and its volume was measured via water displacement to correct soil core volume. Sieved material was weighed, dried at 60°C for 120 h and reweighed.

Within 12 h of sampling, the 5‐cm set of cores from each tree was pooled and homogenized, and 10 g was shaken in 40 ml of 2 M KCl for 1 h, and then filtered and frozen. NO_3_ was analyzed via the vanadium cocktail method (Doane & Horwáth, 2003) and NH_4_ via the phenol‐hypochlorite method (Weatherburn, 1967) using a spectrophotometer (BioTek, Winooski, VT, USA). The remaining soil analyses were performed on fresh, sieved soils by the Centro Agronómico Tropical de Investigación y Enseñanza (CATIE, Turrialba, Costa Rica). The pH was measured in water (1 : 2.5 soil to water). PO_4_ and K were extracted in a modified Olsen solution (pH 8.5) and Ca and Mg were extracted in 1 M KCl. PO_4_ was measured with the molybdenum blue method (Thermo Fisher, Waltham, Ma, USA) and K, Mg and Ca with an atomic absorption spectrophotometer (PerkinElmer, Shelton, CT, USA). Total soil N (N%) was measured with an elemental autoanalyzer (Thermo Finnigan, San Jose, CA, USA). Soil texture fractions were measured via the hydrometer method (Bouyoucos, 1962). Phosphorus fractions were measured via the Hedley fractionation method (Hedley *et al*., 1982) with spectrophotometric measurement after the molybdenum blue method (Thermo Fisher).

### Statistical analyses

To compare overall variation for each fine‐root trait and soil variable, coefficients of variation were calculated by dividing the SD by the mean and multiplying by 100. To partition root trait and soil variable variance among the nested sampling scales, linear mixed effects models for untransformed response variables were fit with random intercepts of the nested sampling scales (i.e. root, tree and site for fine roots; tree and site for soils).

To determine root–soil relationships following standard conventions, linear models were fit for each individual fine‐root trait averaged to the tree level with tree DBH and all soil variables except soil N% as explanatory variables (*n* = 30). Significant and marginally significant explanatory variables were kept in subsequent models until only significant terms remained. Only significant models are reported. After fitting significant models, response variables were Box–Cox transformed to standardize model residuals. Residuals were visually inspected for normality and homoscedasticity. The same process was performed to determine linear root–root relationships (*n* = 90). Bonferroni‐corrected *P*‐values were used to reduce the Type I error rate, whereby the significance threshold of *P* < 0.05 is divided by the number of terms fit to the full model; that is, root–soil significance was considered *P* < 0.005, and root–root significance was considered *P* < 0.00625.

In addition to these common approaches, multivariate statistical analyses were applied to capture the complex natures of both soils and fine roots and to parsimoniously analyze the multivariate data. Before multivariate analyses, non‐normally distributed variables were natural‐log transformed, and then all data were centered and scaled. To examine fine‐root trait coordinations and trade‐offs and to describe soil heterogeneity, principal coordinate analyses were performed (PCA; *n* = 90 and 30, respectively). Variables that explained more variation than average were considered to contribute substantially to principal components. Similarly, the Kaiser–Guttman criterion informed how many principal components were necessary to consider. Redundancy analysis (RDA) was used to identify significant soil drivers of fine‐root trait variation (*n* = 30). Before RDA, collinearity of soil variables was reduced (e.g. total soil carbon was removed), and fine‐root traits that contributed mostly to the third root principal component (i.e. RTD and root branching intensity (RBI)) were removed. The model selection process considered equations indicated by a sequential forward selection procedure and an ecologically informed model. The adjusted *R* ^2^ values, global significance, axis significance and variable significance levels were compared. Significance threshold was considered *P* < 0.05 for multivariate analyses.

Analyses were performed in R 4.3.2 (R Core Team, 2023) with the packages vegan and factominer (Lê *et al*., 2008; Oksanen *et al*., 2025). Figure color palettes come from pnwcolors (Lawlor, 2020).

## Results

### Total variation and spatial scales of variation

**Fig. 3:**
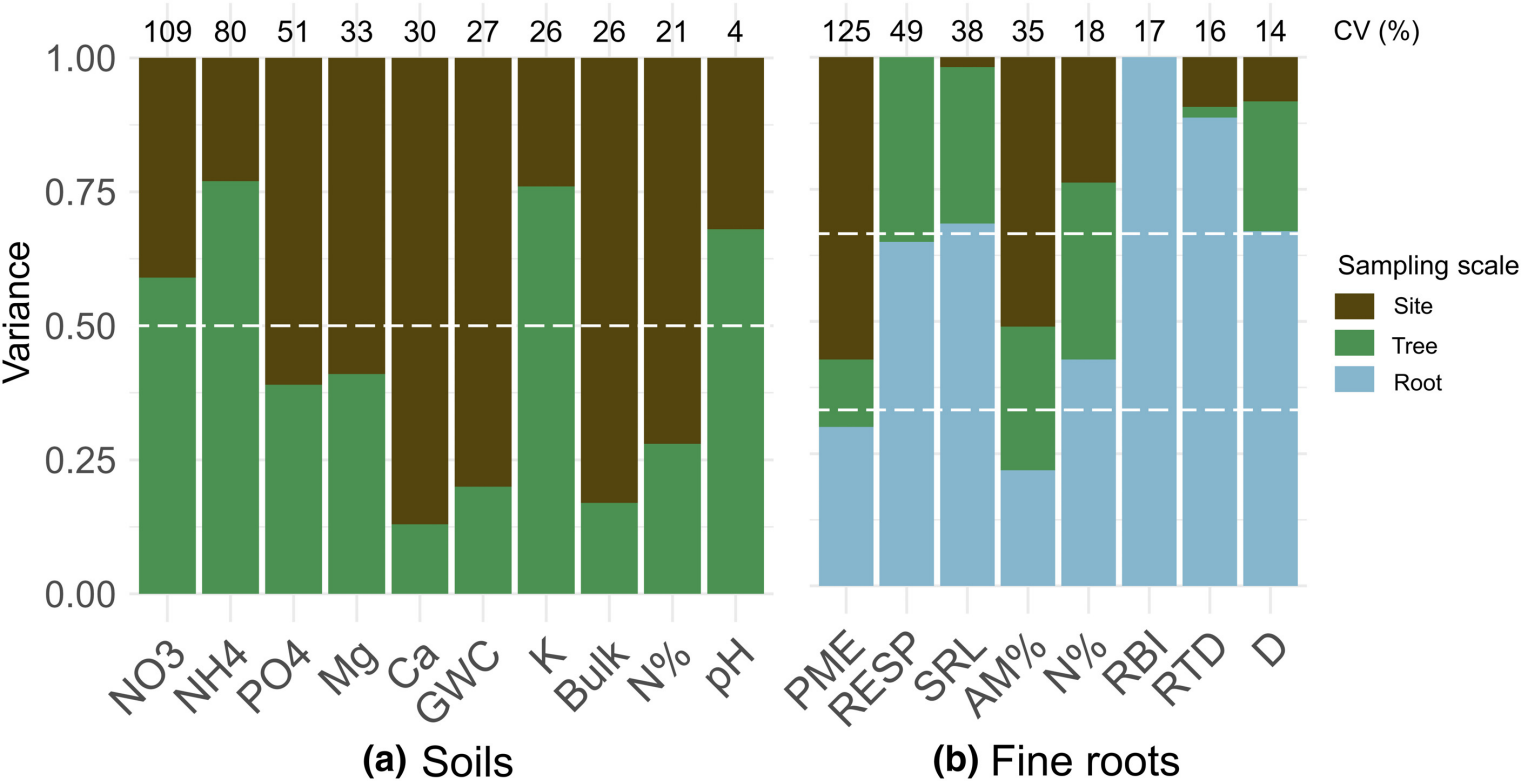
Proportion of total variance explained by each nested sampling scale for raw soil variables (a, *n* = 30) and fine‐root traits of *Handroanthus ochraceus* (b, *n* = 90). Soil variables and fine‐root traits are arranged by descending order of coefficients of variation (CV%) written above each column. The lowest sampling scale includes sampling error. The white dashed lines represents half of the total variance for soils and thirds of the total variance for fine roots. AM%, root arbuscular mycorrhizal colonization intensity; Bulk, soil bulk density; Ca, soil calcium cations; D, root diameter; GWC, soil gravimetric water content; K, soil potassium cations; Mg, soil magnesium cations; N%, root N concentration; N%, soil total N; NH_4_, soil ammonium‐N; NO_3_, soil nitrate‐N; PME, root potential acid phosphomonoesterase activity rate; PO_4_, soil orthophosphate‐P; RBI, root branching intensity; RESP, root respiration rate; RTD, root tissue density; SRL, specific root length.

Total variation and dominant spatial scales of variation differed within soil variables and fine‐root traits of *H. ochraceus* (Dataset S1, Notes S1). Total variation (i.e. the coefficient of variation (CV%)) for soil variables ranged from 4 to 109% (Fig. 3a, Supporting Information Fig. S1). The biologically driven NO_3_ and NH_4_ were the most variable with 109 and 80 CV%, and the dominant spatial scale of variation was within sites rather than among sites (Fig. 3a). By contrast, the rock‐derived nutrients P, Mg and Ca were relatively less variable with 51, 33 and 30 CV%, respectively, and most variance occurred among sites (Fig. 3a). However, the other rock‐derived nutrient, K, mostly varied within sites (Fig. 3a).

**Fig. 4:**
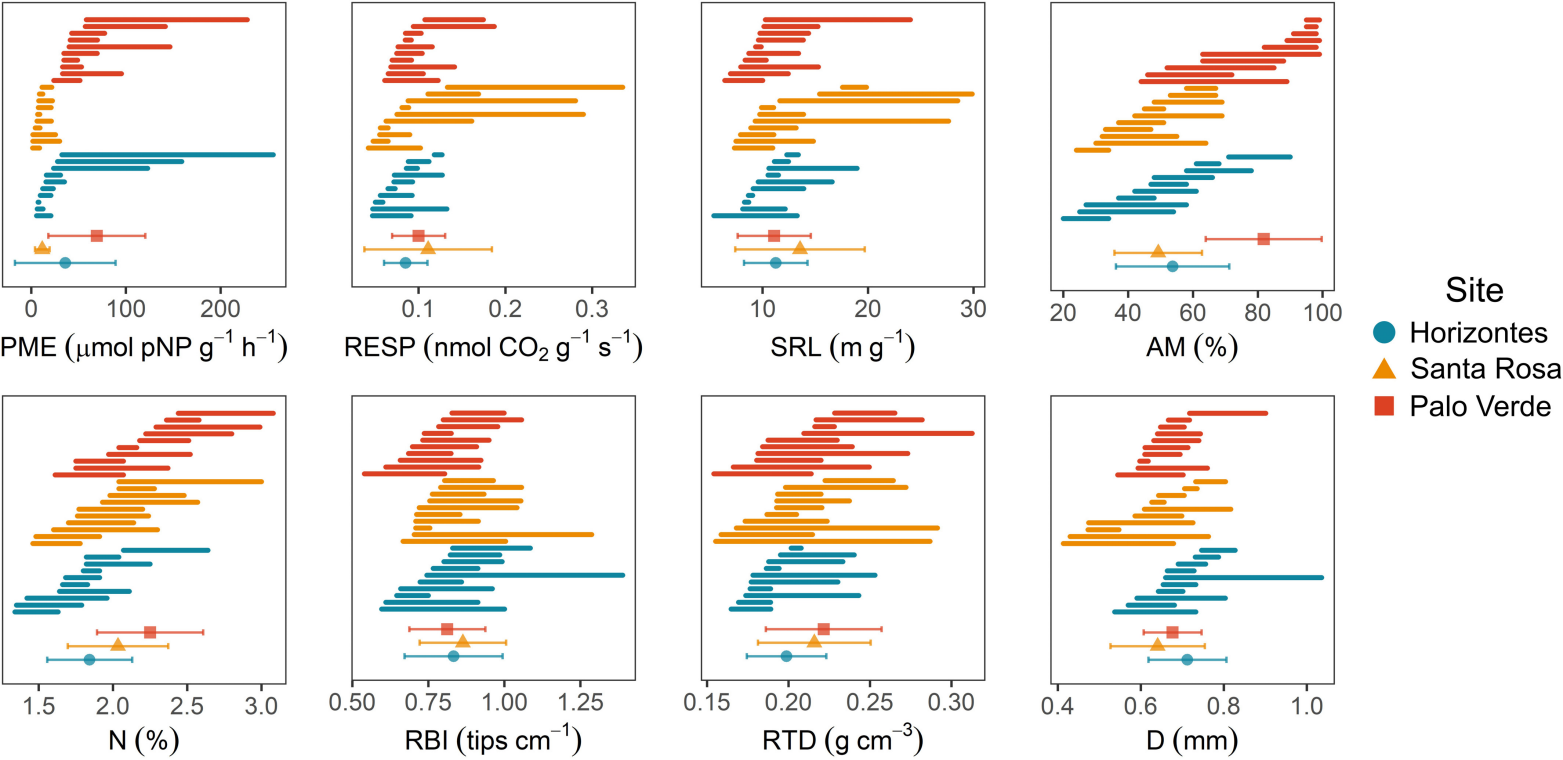
Variation in fine‐root traits across spatial scales for *Handroanthus ochraceus*. Within sites, line segments represent the range of trait values (*n* = 3) for each tree (*n* = 10) arranged by minimum trait value within sites (*n* = 3). Site mean trait values (± SD) are represented by colored shapes at the bottom of each plot. Plots are arranged from left to right, top to bottom, by descending order of coefficients of variation. AM%, root arbuscular mycorrhizal colonization intensity; D, root diameter; N%, root N concentration; PME, root potential acid phosphomonoesterase activity rate; RBI, root branching intensity; RESP, root respiration rate; RTD, root tissue density; SRL, specific root length.

Total variation of fine‐root traits ranged from 14 to 125 CV% (Figs 3b, 4). The physiological traits PME and RESP, the morphological trait SRL and the symbiotic trait AM% varied strongly (125, 49, 38 and 35 CV%, respectively), but whereas PME and AM% varied among sites, RESP and SRL varied within trees (Fig. 3b). The architectural and morphological traits RBI, RTD and D were the least variable (17, 16 and 14 CV%), and mostly varied within trees rather than among trees or sites (Fig. 3b).

### Site characteristics

**Fig. 5:**
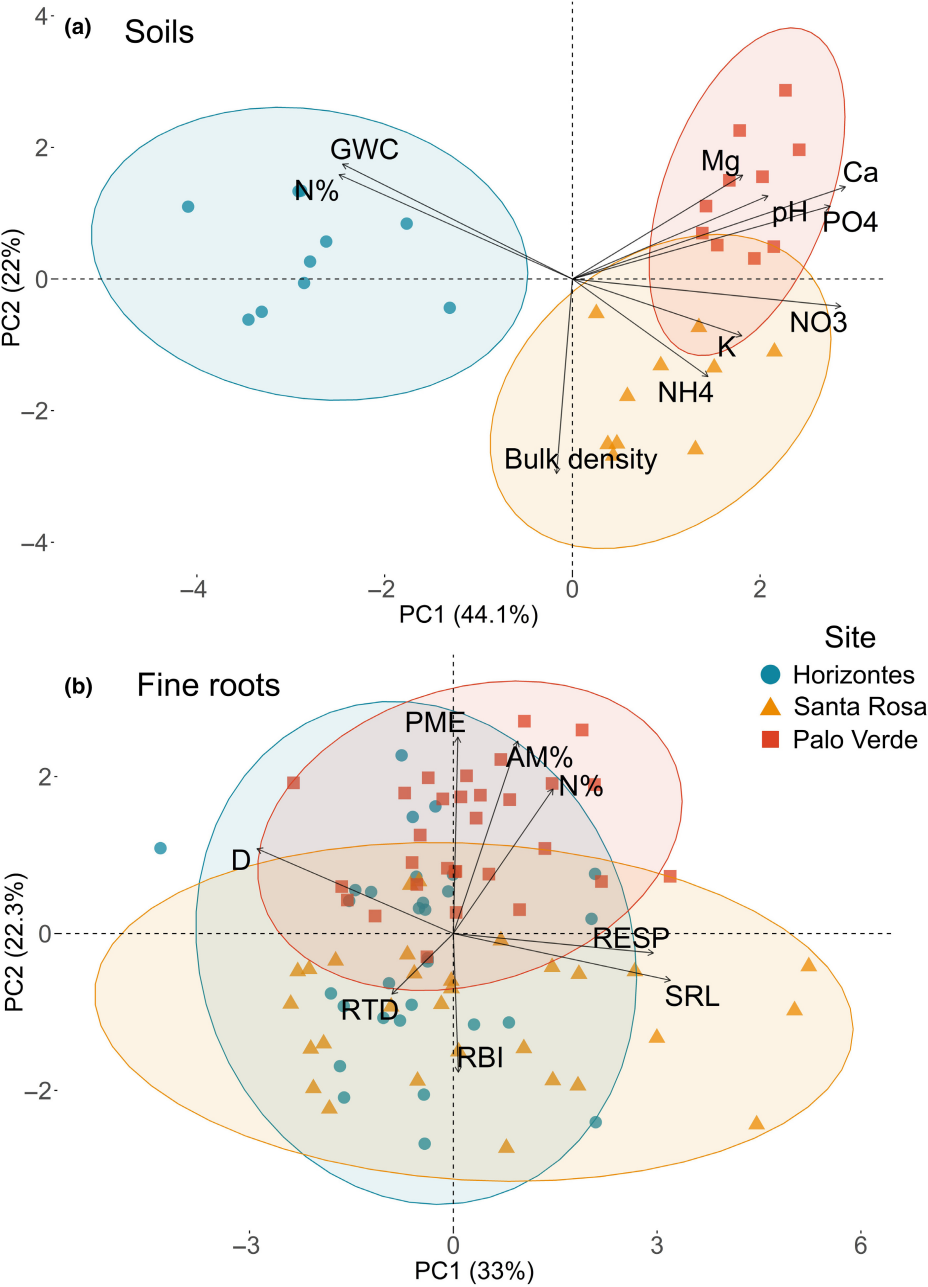
Principal coordinate analyses of soil variables (*n* = 30) and fine‐root traits of *Handroanthus ochraceus* (*n* = 90). Shaded ellipses represent 95% confidence intervals. (a) Soils were primarily distinguished by Ca, NO_3_, PO_4_, N% and GWC (PC1, 44%); secondarily distinguished by bulk density and GWC (PC2, 22%); and tertiarily distinguished by Mg, NH_4_, pH and K (11.2%, not shown). Ca, soil calcium cations; GWC, soil gravimetric water content; K, soil potassium cations; Mg, soil magnesium cations; N%, soil total N; NH_4_, soil ammonium‐N; NO_3_, soil nitrate‐N; PO_4_, soil orthophosphate‐P. (b) Fine roots were primarily distinguished by SRL, RESP, D (PC1, 33%); secondarily distinguished by PME, AM%, N%, RBI (PC2, 22%); and tertiarily distinguished by RTD and RBI (19%, not shown). AM%, root arbuscular mycorrhizal colonization intensity; D, root diameter; N%, root N concentration; PME, root potential acid phosphomonoesterase activity rate; RBI, root branching intensity; RESP, root respiration rate; RTD, root tissue density; SRL, specific root length.

The three sites were edaphically distinct, but Santa Rosa and Palo Verde were more similar to each other than to Horizontes. Sites represented a gradient of nutrient availability opposing sites with high Ca, NO_3_ and PO_4_ against sites with high total N% and GWC; generally, fertility increased from Horizontes, to Santa Rosa, to Palo Verde (principal component 1 (PC1), 44%; Figs 5a, S1). Secondarily, bulk density and GWC were opposed (PC2, 22%; Fig. 5a). Tertiarily, soils high in Mg and NH_4_ were found to oppose soils with higher pH and K (PC3, 11.2%; not shown). Soil texture was similar among sites, but Palo Verde and Santa Rosa soils were clay loams, whereas Horizontes was a sandy loam (Fig. S2; Table S1). Hedley P fractions were also similar among sites, except for Horizontes where total P was about twofold greater due to two‐ to fivefold greater P in the organic NaHCO_3_, organic NaOH and inorganic NaOH extracts (Fig. S3; Table S1).

### Intrinsic drivers of fine‐root traits

Only significant models and predictors are reported, and DBH was not significant in any model. The primary trait coordination and trade‐off observed opposed fine roots with high SRL and RESP against fine roots with a large D (PC1, 33%; Fig. 5b). Secondarily, fine roots with high PME, AM% and N% were opposed with fine roots with a high RBI (PC2, 22%; Fig. 5b). Tertiarily, fine roots with high RTD traded off with high RBI (PC3, 19%; not shown).

**Fig. 6:**
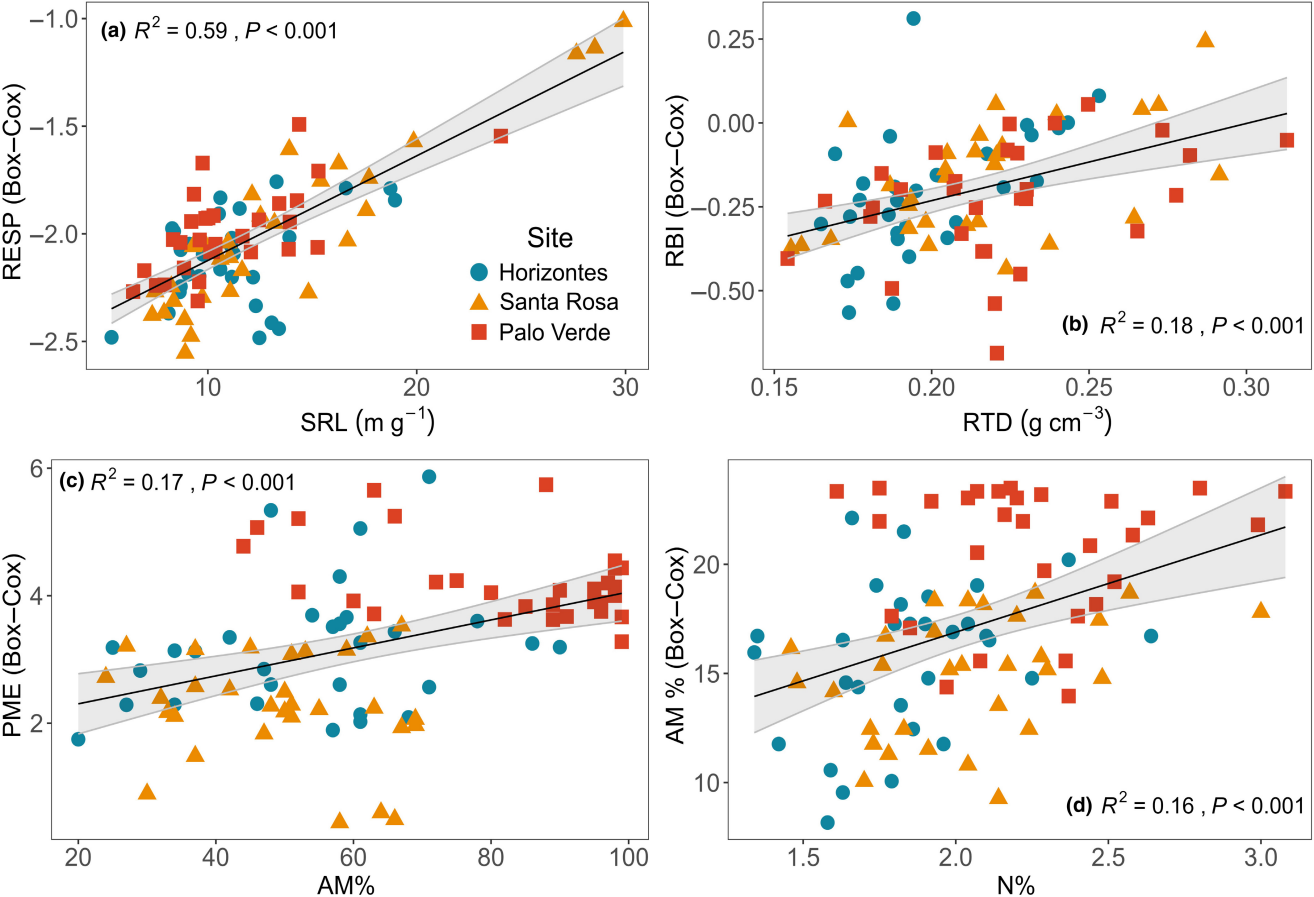
Intrinsic relationships between fine‐root (a) specific root length and respiration rate, (b) tissue density and branching intensity, (c) arbuscular mycorrhizal colonization and phosphomonoesterase activity and (d) root nitrogen concentration and arbuscular mycorrhizal colonization for *Handroanthus ochraceus* with fitted linear models in black and 95% confidence intervals in gray (*n* = 30). Only significant relationships based on ANOVA tests with a Bonferroni‐corrected significance threshold are shown (*P* < 0.00625). AM%, root arbuscular mycorrhizal colonization intensity; N%, root N concentration; PME, root potential acid phosphomonoesterase activity rate; RBI, root branching intensity; RTD, root tissue density.

Root–root linear analyses confirmed that morphological traits are interrelated, but notably identified relationships among morphological, chemical, physiological and symbiotic traits. Root respiration rate increased with SRL, explaining over half the variation in the trait (*R* ^2^ = 0.59, *P* < 0.001; Fig. 6a; Table S2). Root branching intensity also positively correlated with RTD (*R* ^2^ = 0.18, *P* < 0.001; Fig. 6b; Table S2). As AM% increased, so did PME (*R* ^2^ = 0.17, *P* < 0.001; Fig. 6c; Table S2). Nitrogen concentration and AM% were positively correlated (*R* ^2^ = 0.16, *P* < 0.001; Fig. 6d; Table S2), and AM% was also positively correlated with N% (*R* ^2^ = 0.16, *P* < 0.001; Table S2).

### Edaphic drivers of fine‐root function

**Fig. 7:**
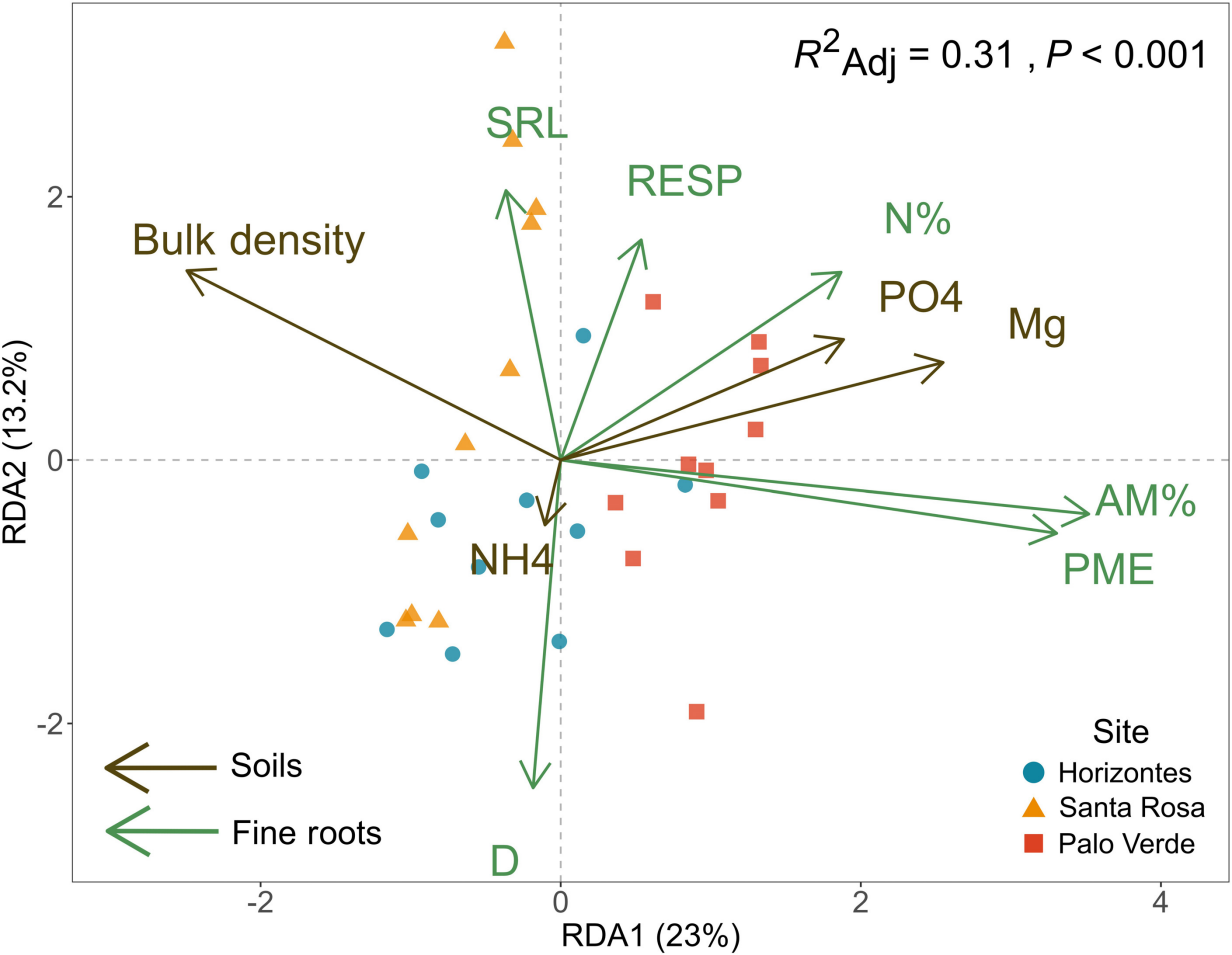
Redundancy analysis showing all significant edaphic influences on fine‐root traits of *Handroanthus ochraceus*, averaged to the tree level and colored by site (scaling = 2, *n* = 30). The first axis of the redundancy analysis (RDA1) shows that AM% and PME positively respond to soil Mg and PO_4_ and negatively respond to bulk density (*P* < 0.05). To a lesser degree, RDA2 shows that D positively responds to soil NH_4_, whereas SRL, RESP and N% respond negatively (*P* < 0.05). RTD and RBI were not included. AM%, root arbuscular mycorrhizal colonization intensity; D, root diameter; Mg, soil magnesium cations; N%, root N concentration; NH_4_, soil ammonium‐N; PME, root potential acid phosphomonoesterase activity rate; PO_4_, soil orthophosphate‐P; RBI, root branching intensity; RESP, root respiration rate; RTD, root tissue density; SRL, specific root length.

Only significant models and predictors are reported, and DBH was not significant in any model. Redundancy analysis identified multivariate soil drivers of the multidimensional fine‐root trait space (*R* ^2^_adj_ = 0.31, *P* < 0.001; Fig. 7). Fine‐root PME and AM% increased as soil PO_4_ and Mg increased and as bulk density decreased (RDA1, 23%, *P* < 0.05; Fig. 7). To a lesser extent, fine‐root SRL, RESP and N% increased and D decreased as soil NH_4_ decreased (RDA2, 13%, *P* < 0.05; Fig. 7).

**Fig. 8:**
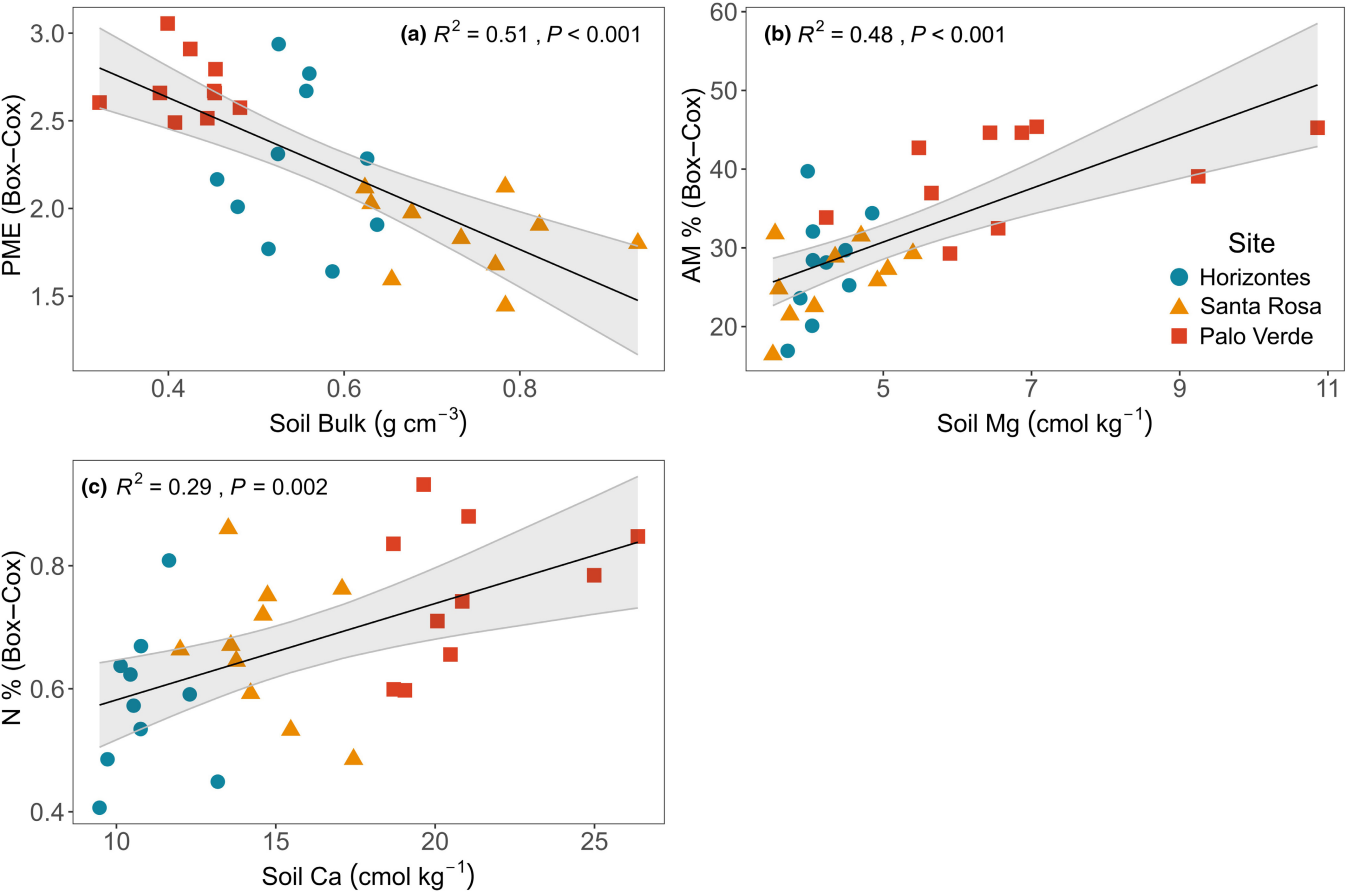
Significant relationships between individual fine‐root traits of *Handroanthus ochraceus* and soil drivers with fitted linear models in black and 95% confidence intervals in gray (*n* = 30): (a) soil bulk density and root phosphomonoesterase activity rate, (b) soil magnesium cation concentration and root arbuscular mycorrhizal colonization, (c) soil calcium cation concentration and root nitrogen concentration. Only significant relationships based on ANOVA tests with a Bonferroni‐corrected significance level are shown (*P* < 0.005). AM%, root arbuscular mycorrhizal colonization intensity; Ca, soil calcium cations; Mg, soil magnesium cations; N%, root N concentration; PME, root potential acid phosphomonoesterase activity rate.

As in the multivariate analysis, linear models showed that PME declined as soil bulk density increased (*R* ^2^ = 0.51, *P* < 0.001; Fig. 8a; Table S3) and AM% increased with soil Mg (*R* ^2^ = 0.48, *P* < 0.001; Fig. 8b; Table S3). One linear trend was not detected in the multivariate analysis. Root N% increased with soil Ca (*R* ^2^ = 0.29, *P* = 0.002; Fig. 8c; Table S3).

## Discussion

Work across coarse soil gradients has thus far largely failed to identify edaphic drivers of the high variation observed for fine‐root traits. Given the complexity of the multidimensional soil mosaic, where each soil property can vary at distinct spatial scales and independently from other properties, it is important to spatially couple fine‐root and soil samples to uncover their relationships. In this way, root–soil relationships can be examined across multiple spatial or biological scales so that fine‐scale relationships especially are less likely to be overlooked, and so each soil property can be evaluated at comparable resolutions. Using such a sampling scheme, our data suggest that the two main fine‐root trait trade‐offs and coordinations of *H. ochraceus* were shaped by soil heterogeneity. We found evidence that the largest source of fine‐root trait variation as defined by the first principal component of the PCA, namely the trade‐off between large D and high SRL and RESP, was driven by fine‐scale variation of NH_4_ within sites or potentially within a single tree's root system (Figs 3b, 5b, 7). The second largest source of fine‐root trait variation, namely the coordination between AM% and PME in the second principal component of the PCA, was driven by coarse‐scale heterogeneity in bulk density and Mg largely among sites (Figs 3b, 5b, 7). These findings suggest that fine‐scale soil heterogeneity may substantially shape fine‐root function and that a single species can respond to multiple spatial scales of soil heterogeneity at once.

### Spatial scales of variation

We hypothesized that a disproportionate amount of variance would occur within trees for fine roots and within sites for soil variables (i.e. greater than one‐third and one‐half of total variance, respectively), equating to the finest sampling scale. Spatial scales of variation vastly differed among fine‐root traits (Fig. 3b), but similar to prior studies, much of the variation occurred at the finest sampling scale (Waring *et al*., 2016; Defrenne *et al*., 2019; Weemstra *et al*., 2021). Five of eight fine‐root traits largely varied within trees (> 1 m), indicating high trait plasticity within individuals (Fig. 3b). For two other fine‐root traits, variation was largely observed among sites (15–60 km), whereas root N% variation was more evenly split among sites, trees (3–60 m) and fine roots (Fig. 3b). Variation within sites was also important, but usually less substantial than among sites or within trees (Fig. 3b).

The spatial scales of soil variation differed by the apparent drivers behind their spatial structuring. Rock‐derived nutrients, such as PO_4_, Mg and Ca, mostly varied across coarse spatial scales, perhaps representing differing parent materials, ages or climatic conditions among sites (Fig. 3a). However, K varied largely within sites (Fig. 3a). Relatively small patches of K were found in an old‐growth Panamanian forest and perhaps represent plant restructuring of this key macronutrient under canopies through litter inputs and root uptake and exudation  (i.e. Zinke effects; Yavitt *et al*., 2009; Waring *et al*., 2015). Ammonium and NO_3_ also varied within sites, potentially because they are also structured by Zinke effects and probably structured at even finer spatial scales by microbial activity (Waring *et al*., 2015, 2016; Akana *et al*., 2023).

### Intrinsic drivers of fine‐root function

We hypothesized that most fine‐root trait variation would be explained by a coordination between D and AM% that would trade off with SRL and PME; the second largest source of variation would be explained by a coordination between N% and RESP that would trade off with RTD. Despite these trade‐offs being primarily found among species, we expected them to manifest within a single species, indicating that these trade‐offs can hold across scales due to physiological constraints. Intraspecific fine‐root trait coordinations and trade‐offs identified by PCA were similar to our hypotheses with some notable differences that may indicate emerging tropical trends. Differing from Bergmann *et al*.'s (2020) findings, fine root D and SRL represented the primary trade‐off but were not aligned with AM%; N% was part of the secondary trade‐off, but not with RTD (Fig. 5b). Instead, D traded off with fine roots with high SRL and RESP, and independently, fine roots with high N%, AM% and PME traded off with high RBI (Fig. 5b).

The relationship between SRL and RESP was very strong, suggesting that SRL could be a good candidate for a RESP proxy intraspecifically (Fig. 6a; Table S2). Interspecifically, a similarly strong and consistent relationship was found across 252 plant species from subtropical and temperate China (Liang *et al*., 2023). We also found that RBI increased with RTD, which could indicate that soil exploration was maintained to some extent (i.e. by increasing RBI) despite increasing RTD, which would increase root construction costs but also confer greater biotic or mechanical resistance (Fig. 6b).

Contrary to predictions, AM% was not related to D or other morphological traits. Instead, our findings suggest that AM% and root N% were related, and in turn, PME and AM% were related, indicating interactions between symbiotic investment, root chemistry and physiological activity (Figs 6c,d, 7; Table S2). Several other recent tropical studies have found that AM% aligns with fine‐root traits other than D; instead, AM% appears to coordinate with PME and N% and trade off with RBI in PCAs (Guilbeault‐Mayers *et al*., 2020; Yaffar *et al*., 2021; Liang *et al*., 2023; Marcellus *et al*., 2024), but see Han & Zhu (2021). These findings suggest that tropical fine‐root AM% may be decoupled from root morphology and rather tends to align with N% on the so‐called conservation gradient (RTD and N%) in addition to PME.

Our findings of a positive relationship between AM% and PME, in addition to their alignment in PCAs of other tropical studies, suggest that AM fungi may be contributing to PME contrary to the prevailing theory that these fungi do not produce such enzymes (Guilbeault‐Mayers *et al*., 2020; Yaffar *et al*., 2021; Marcellus *et al*., 2024). Greater AM% could also increase the probability that mineralized PO_4_ will be absorbed by hyphae and transferred to the tree.

The directionality between root N% and AM% relationships is unclear in the present study, but may indicate that roots become enriched in N when colonized by mycorrhizal fungi (Fig. 6d). Nitrogen enrichment could be caused by improved N uptake and assimilation caused by AM fungal impacts on plant N metabolism and transporters, as experimental works have shown for woody *Catalpa bungei* and *Camellia sinensis* plants (Chen *et al*., 2023; Wu *et al*., 2024).

### Edaphic drivers of fine‐root function

High‐resolution, spatially coupled sampling of fine roots and soils was a powerful approach to address hypotheses about root‐soil relationships. We hypothesized that P would drive most fine‐root trait responses, causing downregulation of traits, such as PME, AM%, and/or SRL as P availability increased, meaning individual traits would become more conservative as limitation lifted; we expected that increasing availability of NH_4_ and NO_3_ would decrease SRL and increase RTD. The two fine‐root trait coordinations and trade‐offs observed with PCA were found to be driven by soil variables with RDA. Briefly, RDA identifies statistically significant relationships between soil variables and fine‐root traits in multivariate space. In both PCA and RDA, the amount of variation explained by an axis corresponds to the axis order. The primary RDA axis was driven by bulk density, Mg and PO_4_, and influenced fine‐root PME and AM% (Fig. 7). Potential acid phosphomonoesterase activity rate and AM% were involved in the secondary fine‐root coordination of the PCA in addition to N% and RBI (Fig. 5b). The secondary RDA axis was driven by NH_4_ and influenced D, SRL, RESP and N% (Fig. 7). Diameter, SRL and RESP were involved in the primary fine‐root coordination of the PCA, whereas N% contributed to the secondary coordination of the PCA (Fig. 5b). Bivariate linear analyses confirmed two root–soil multivariate relationships and identified a new one: Bulk density negatively affected PME, soil Mg positively influenced AM%, and root N% was driven by Ca (Figs 7, 8).

Multivariate analyses identified that the secondary axis of variation for fine roots (i.e. AM% and PME) responded positively to soil Mg and PO_4_ and negatively to bulk density (Figs 5b, 7). Based on their bivariate relationship, it appears that PME was mostly negatively responding to bulk density (Fig. 8a). Bulk density could influence enzyme activity through its impact on soil sorption potential and organic matter. Higher bulk density means there are more surfaces for enzymes and substrates to sorb to in a given volume, so mineralization and plant uptake are less likely (Holz *et al*., 2019). As bulk density increases, soil organic matter can decline, meaning a reduction in the enzyme's substrate leading to a downregulation of enzyme production (Cabugao *et al*., 2021). Based on this reasoning, *H. ochraceus* may have reduced PME investment as soil properties became less favorable. It is also possible that PME increased with soil PO_4_ (Fig. 7), although this was not expected based on interspecific trends in other tropical trees that showed the opposite (Ushio *et al*., 2015; Guilbeault‐Mayers *et al*., 2020; Cabugao *et al*., 2021). The mechanistic links are unclear, but it could represent an intraspecific trend that opposes interspecific expectations (Laughlin *et al*., 2017; Anderegg *et al*., 2018; Anderegg, 2023). For example, an analysis performed on a large leaf trait dataset found some relationships of the leaf economics spectrum held independently of taxonomic and ecological scales, but other trade‐offs could be reversed when comparing inter‐ vs intraspecific variation (Anderegg *et al*., 2018).

Soil exchangeable Mg drove increased mycorrhizal colonization of *H. ochraceus* roots (Figs 7, 8b). Although we did not have *a priori* hypotheses for fine‐root responses to soil cations, there is in fact diverse evidence to support the importance of Mg in the arbuscular mycorrhizal symbiosis and for tropical trees generally. Magnesium fertilization has been found to increase AM% in controlled studies on tomato and maize (Gryndler *et al*., 1992; Ardestani *et al*., 2011; Liu *et al*., 2023). In the Amazon, an *in situ* cation fertilization that applied a mixture of Mg, Ca and K resulted in increased AM% of community‐scale fine‐root samples (Lugli *et al*., 2021). This finding agrees with our results and reinforces the potentially important role of Mg in regulating the arbuscular mycorrhizal symbiosis (Lugli *et al*., 2021).

That Mg has substantial impacts on AM% in the field and the greenhouse, and in community‐level samples and diverse species‐specific samples, implicates Mg as a key regulator of the AM symbiosis. Recent experimental works have suggested that Mg fertilization could improve AM% by optimizing sucrose translocation to roots, thereby provisioning symbionts (Peng *et al*., 2018; Liu *et al*., 2023). Mechanistically, phloem sucrose loading can be an active transport process mediated by a sucrose‐H^+^ symporter that is dependent on a proton gradient created by ATP, while the phosphorylation of ADP to form ATP is dependent on Mg‐ATPase; as Mg declines, starch and sugar accumulate in leaves and dwindle in roots, which restricts symbiont provisioning (Hermans *et al*., 2005; Zhang & Turgeon, 2018; Tian *et al*., 2021; Liu *et al*., 2023). However, we are unsure of the exact phloem‐loading mechanism for *H. ochraceus* and most trees load phloem passively via sucrose concentration gradients between leaves and phloem (Zhang & Turgeon, 2018). Magnesium may have alternatively improved photoassimilation, being the central atom of Chl*a* and Chl*b* and the activator of the RuBisCO enzyme, allowing greater symbiont provisioning (Tränkner *et al*., 2018; Tian *et al*., 2021). Magnesium and other cations have also been found to influence tropical tree species' distributions to similar extents as N and P, indicating that cations may substantially shape tropical tree biogeography and fine‐root function (John *et al*., 2007; Kaspari & Powers, 2016). Calcium drove an increase in root N%, perhaps representing a shift to a more acquisitive fine‐root trait value as soil Ca limitation lifted (Fig. 8c).

Multivariate analyses also identified soil NH_4_ as the driver for the primary axis of fine‐root trait variation (i.e. D, SRL, RESP of PC1 and N% of PC2; Figs 5b, 7). As NH_4_ increased, SRL, RESP and N% declined and D increased, but this relationship was relatively weak as demonstrated by the arrow length of NH_4_ (Fig. 7). This trait trade‐off may reflect increased soil exploration and physiological activity to maintain N acquisition. Although a decrease in root N% as soil NH_4_ increased was unexpected, since root nutrient concentrations often increase with soil nutrients, it may reflect altered N assimilation pathways. Plants absorb NO_3_ and NH_4_ through specific transporters, but whereas NH_4_ can be assimilated directly, NO_3_ assimilation requires two additional enzymes, nitrate reductase and nitrite reductase (Kishorekumar *et al*., 2020). Mechanistically, increasing NH_4_ availability may have downregulated the expression of both enzymes, leading to lower RESP and potentially also N% (Wany *et al*., 2019; Kishorekumar *et al*., 2020). Simultaneously, SRL declines could indicate a reduced need for soil exploration. Decreased RESP rates could also reflect both lower N% and SRL due to reduced construction and maintenance respiration of fine roots and endoenzymes (Kishorekumar *et al*., 2020; Liang *et al*., 2023).

We sampled soils and fine roots at one time point, and thus did not capture seasonal variation in soil nutrient availability, which is known to occur in seasonally dry tropical forests (Roy & Singh, 1995; Waring *et al*., 2021). However, a recent study that quantified soil nutrients, microbial biomass and enzyme activities in four diverse neotropical forests – including one of the sites used in this study – found that variation within sites was as large or larger than seasonal variation for all parameters (Waring *et al*., 2021). Although our design focused specifically on spatial rather than temporal variation, we may have still captured the dominant source of variability in soil characteristics most likely to influence fine‐root traits.

### Effects of spatial scales of variation on root–soil relationships

We found support for our hypothesis that root–soil relationships are strongest when spatial scales of variation in fine‐root traits and soil variables are adequately captured and spatially matched. Specifically, fine‐root traits that largely varied among sites were strongly driven by soil variables that also largely varied among sites: soil Mg, PO_4_ and bulk density, and root AM% and PME (Figs 3a,b, 7). By contrast, the root–soil relationship between fine‐root traits that largely varied within trees was relatively weakly driven by a soil variable that largely varied within sites: soil NH_4_ and root D, SRL, RESP and N% (Figs 3b, 7). Edaphic resolution for NH_4_ was possibly lost by pooling soil cores collected adjacent to each fine‐root sample to the tree level, which would obscure responses if inorganic N forms were highly heterogeneous within the spatial scale of a single tree's root system. In a temperate forest study, soil NO_3_ and NH_4_ were substantially heterogeneous at spatial scales < 1 m, with spatial autocorrelation only observed at spatial scales < 2.3 m (Akana *et al*., 2023). Considering that the trade‐off between D, SRL and RESP explained the greatest amount of variation in fine‐root data as PC1, it is possible that this root–soil relationship would have been the strongest if soils were characterized at a higher resolution (Fig. 5b). However, due to the loss of soil resolution that included putatively important variation, this relationship was weaker and led to its classification as RDA2 (Fig. 7).

In summary, edaphic properties drove fine‐root trait function in *H. ochraceus* at two spatial scales simultaneously: among sites (15–60 km) and within sites (3–60 m). The high level of within‐tree variation, especially in traits found to respond to NH_4_, suggests that edaphic properties at finer scales (*c*. 1 m) may also have influenced trait expression within individual trees, but this could not be tested here. Future work should prioritize even finer soil spatial resolution, seeking to directly spatially couple a soil sample to any fine‐root samples collected. This practice could help to capture these root–soil relationships at multiple spatial scales and possibly reduce bias against root–soil relationships involving soil properties that predominately vary at fine scales.

### Conclusions

Our high‐resolution sampling and multivariate analysis were powerful approaches to discover edaphic and intrinsic fine‐root relationships. We identified several strong root–soil and root–root relationships that help us to better understand the constraints and drivers of fine‐root function. While further testing is required to determine whether this high‐resolution sampling approach is applicable across many species, for example due to greater trait diversity and nonindependence structures of data, the strength of the root–soil relationships found here is promising.

Some of the relationships identified here could be incorporated into models to better represent the edaphic drivers of fine‐root function and its impacts on carbon and mineral nutrient cycling. We found additional support for a strong trait relationship between SRL and RESP, which can be used to model the plant's role in the carbon cycle (Liang *et al*., 2023; Marcellus *et al*., 2024); SRL is also one of the few fine‐root traits commonly included in global and vegetation models (Cusack *et al*., 2024). The driving role of Mg on AM% is an especially strong candidate for a root–soil relationship; although models do not currently include Mg maps, parent material and/or P maps could perhaps be used as proxies for rock‐derived nutrients generally in further exploration. Although it currently seems unlikely that soil variable heterogeneity at fine scales could be represented in global and vegetation models, knowing the directionality of root–soil relationships can inform responses to coarse‐scale heterogeneity. For example, although N%, SRL and RESP increased as NH_4_ decreased at fine spatial scales, this response could possibly be assumed to apply at coarse scales in models. However, models may simply not be able to represent the important fine‐root responses that occur at these finer scales, in which case, modelers might instead consider representing these traits more flexibly (Dantas de Paula *et al*., 2021; Rius *et al*., 2023).

Interestingly, AM% and PME were aligned and were decoupled from root morphology, meaning that many intensities of symbiotic and enzyme investment can be expressed for a given root morphology within a species, increasing their potential functional breadth. Instead of AM% being driven by D, we found that fine roots with higher AM% expressed higher PME, while fine roots with higher N% had higher AM%. These intrinsic relationships suggest that arbuscuar mycorrhizal fungi influence N and P nutrition, increasing N metabolism and enhancing PO_4_ mineralization in tropical trees. The flexibility of these trait relationships with respect to fine‐root morphology could also potentially be incorporated into models.

Practically, the alignment of spatial scales of variation between significant root–soil relationships found here could be used to guide both sampling methodologies (which could prioritize direct spatial coupling of root and soil samples) and predictions of fine‐root functional responses to edaphic properties (which likely reflect the underlying spatial heterogeneity of relevant soil properties). The role of cations observed here supports the calls of Kaspari & Powers (2016) to embrace all essential elements in biogeography; we suggest that plant ecophysiologists also consider a more diverse array of nutrients and their relevant chemical forms when investigating plant–soil interactions, especially in highly weathered ecosystems.

## Competing interests

None declared.

## Author contributions

CD and FMS conceived and designed the study. CD and LM performed the data collection and analyses. CD performed statistical analyses. CD, FMS, JSP and LM all interpreted results and contributed to the manuscript – writing led by CD.

## Disclaimer

The New Phytologist Foundation remains neutral with regard to jurisdictional claims in maps and in any institutional affiliations.

## Supporting information


**Dataset S1** Raw fine‐root and soil data collected and analyzed in this study.

**Notes S1** Explanations of column names and units in Dataset S1.

**Fig. S1** Raw soil data ranges for sites in Guanacaste, Costa Rica.
**Fig. S2** Mean soil texture fractions for each site in Guanacaste, Costa Rica.
**Fig. S3** Mean Hedley phosphorus fractions for soil samples pooled between the two nearest trees in each site in Guanacaste, Costa Rica.
**Table S1** Soil texture and phosphorus fraction means ± SD for each study site in Guanacaste, Costa Rica.
**Table S2** Significant root–root linear models for *Handroanthus ochraceus*.
**Table S3** Significant root–soil linear models for *Handroanthus ochraceus*.
Please note: Wiley is not responsible for the content or functionality of any Supporting Information supplied by the authors. Any queries (other than missing material) should be directed to the *New Phytologist* Central Office.

## Data Availability

The data collected in this study are included in Dataset S1. Explanations of the column names, units and some methods are included in Notes S1. Data will also be openly available in v.4.0 of the Fine Root Ecology Database (https://roots.ornl.gov/), identifiable with this paper's citation.
