# Supplementary material for: Strong scale‐dependent relationships between fine‐root function and soil properties uncovered with spatially coupled sampling
Source: New Phytol. 2025 Apr 29;246(6):2506–21. doi: 10.1111/nph.70143 (PMC12095980; doi:10.1111/nph.70143)
Supplement: Supplementary file 3 — Fig. S1 Raw soil data ranges for sites in Guanacaste, Costa Rica. Fig. S2 Mean soil texture fractions for each site in Guanacaste, Costa Rica. Fig. S3 Mean Hedley phosphorus fractions for soil samples pooled between the two nearest trees in each site in Guanacaste, Costa Rica. Table S1 Soil texture and phosphorus fraction means ± SD for each study site in Guanacaste, Costa Rica. Table S2 Significant root–root linear models for Handroanthus ochraceus. Table S3 Significant root–soil linear models for Handroanthus ochraceus. Please note: Wiley is not responsible for the content or functionality of any Supporting Information supplied by the authors. Any queries (other than missing material) should be directed to the New Phytologist Central Office. [file NPH-246-2506-s002.pdf]

New Phytologist Supporting Information

Title: Strong scale-dependent relationships between fine-root function and soil properties uncovered with spatially-coupled sampling

Authors: Caroline Dallstream, Lola Milder, Jennifer S Powers, Fiona M Soper

Acceptance date: 25 March 2025

Table S1. Soil texture and phosphorus fraction means ± SD for each study site (n = 5) in Guanacaste, Costa Rica. Here, Pi represents inorganic phosphorus fraction and Po represent organic fractions. See Fig. **S1** for stacked bar chart of Hedley phosphorus fractions.

Soil Variable	Horizontes	Santa Rosa	Palo Verde
Sand (%)	52.8 ± 1.1	36.5 ± 4.0	36.4 ± 7.9
Silt (%)	32.8 ± 1.8	31.7 ± 1.4	28.6 ± 1.8
Clay (%)	14.4 ± 0.9	31.8 ± 3.7	35.0 ± 6.6
Pi NaHCO3 (mg kg^{-1})	14.0 ± 2.3	10.5 ± 2.1	14.4 ± 2.8
Po NaHCO3 (mg kg^{-1})	28.2 ± 3.2	12.4 ± 3.0	10.7 ± 1.9
Pi NaOH (mg kg^{-1})	61.6 ± 7.5	15.6 ± 1.4	12.2 ± 2.4
Po NaOH (mg kg^{-1})	272.2 ± 22.3	123.6 ± 15.5	119.2 ± 13.3
Pi HCl (mg kg^{-1})	7.5 ± 2.2	12.1 ± 2.5	5.2 ± 1.4
P residual (mg kg^{-1})	108.0 ± 8.9	73.2 ± 12.5	71.8 ± 6.3
P fractions sum (mg kg^{-1})	489.8 ± 41.3	247.4 ± 34.0	231.6 ± 16.7

Table S2. Significant root-root linear models for *Handroanthus ochraceus*. Each response trait was tested against all other root traits and tree DBH, and significance level was determined with ANOVA and Bonferroni-corrected p-values for multiple comparisons (significance p-value < 0.00625). Response variables were Box-Cox transformed (using lambda) to normalize residuals. For each model, the lambda, R^2 value, p-value and degrees of freedom (DF) are listed, and the estimate and standard error of explanatory variables are listed.

Response Trait (Box-Cox)	Explanatory Root Variables	Box-Cox Lambda	R^2	p-value	DF	Estimate	SE
Diameter	Specific Root Length	-1.31	0.95	<0.001	2	-0.07	0.002
	+ Root Tissue Density					-3.94	0.21
Respiration Rate	Specific Root Length	0.14	0.59	<0.001	1	0.05	0.004
Root Branching Intensity	Root Tissue Density	-0.34	0.18	<0.001	1	2.30	0.52
Phosphomonoesterase Activity	Mycorrhizal Colonization	0.02	0.17	<0.001	1	0.02	0.005
Mycorrhizal Colonization	Root N Concentration	0.59	0.16	<0.001	1	4.46	1.1
Root N Concentration	Mycorrhizal Colonization	-0.06	0.16	< 0.001	1	0.003	0.0008

Table S3. Significant root-soil linear models for *H. ochraceus*. Each response trait was tested against all other soil variables and tree DBH, and significance level was determined with ANOVA and Bonferroni-corrected p-vaues for multiple comparisons (significance p-value < 0.005). Response variables were Box-Cox transformed (using lambda) to normalize residuals. For each model, the lambda, R^2 value, p-value and degrees of freedom (DF) are listed, and estimate and standard error of explanatory variables are listed.

Response Trait (Box-Cox)	Explanatory Soil Variable	Lambda	R^2	p-value	DF	Estimate	SE
Phosphomonoesterase Activity	Bulk Density	-0.22	0.51	<0.001	1	-2.16	0.40
Mycorrhizal Colonization	Magnesium	0.79	0.48	<0.001	1	3.41	0.66
Root Nitrogen Concentration	Calcium	-0.14	0.29	<0.001	1	0.02	0.005

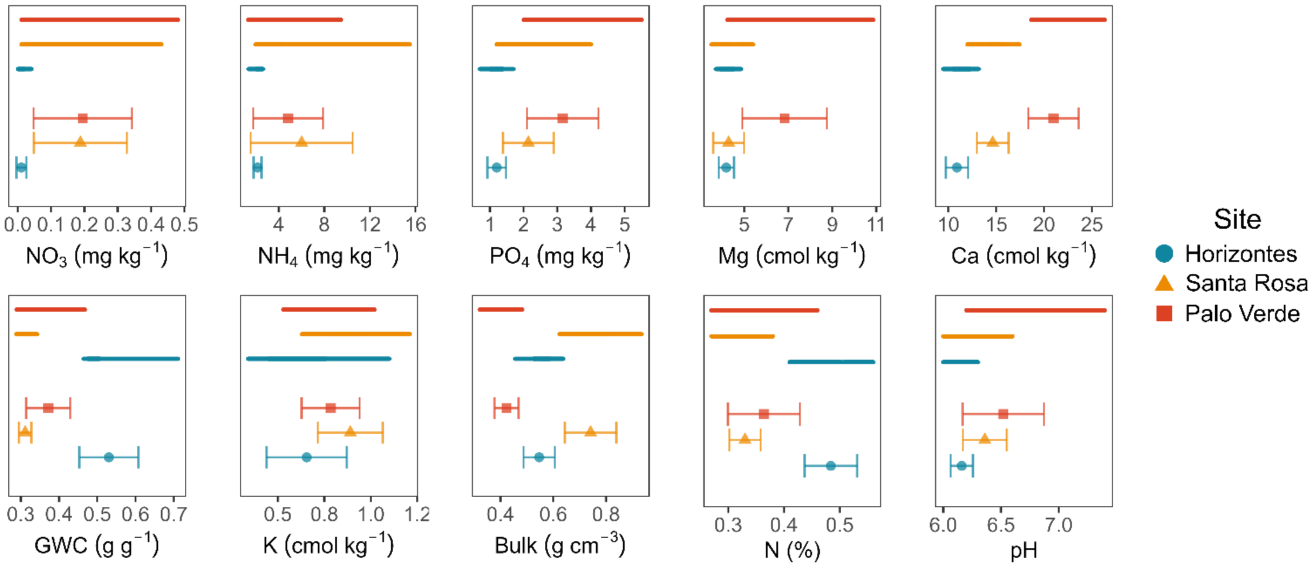

Figure S1. Raw soil data ranges for sites in Guanacaste, Costa Rica. Each line represents the range of values observed within each site (n = 3) for soils collected around trees (n = 10). Colored shapes at the bottom represent mean values (± SD) for each site. Plots are arranged from left to right, top to bottom by descending order of coefficients of variation. Bulk – bulk density, Ca – soil calcium cations, GWC – soil gravimetric water content, K – soil potassium cations, Mg – soil magnesium cations, N% – soil total N, NH_4 – soil ammonium-N, NO_3 – soil nitrate-N, PO_4 – soil orthophosphate-P.

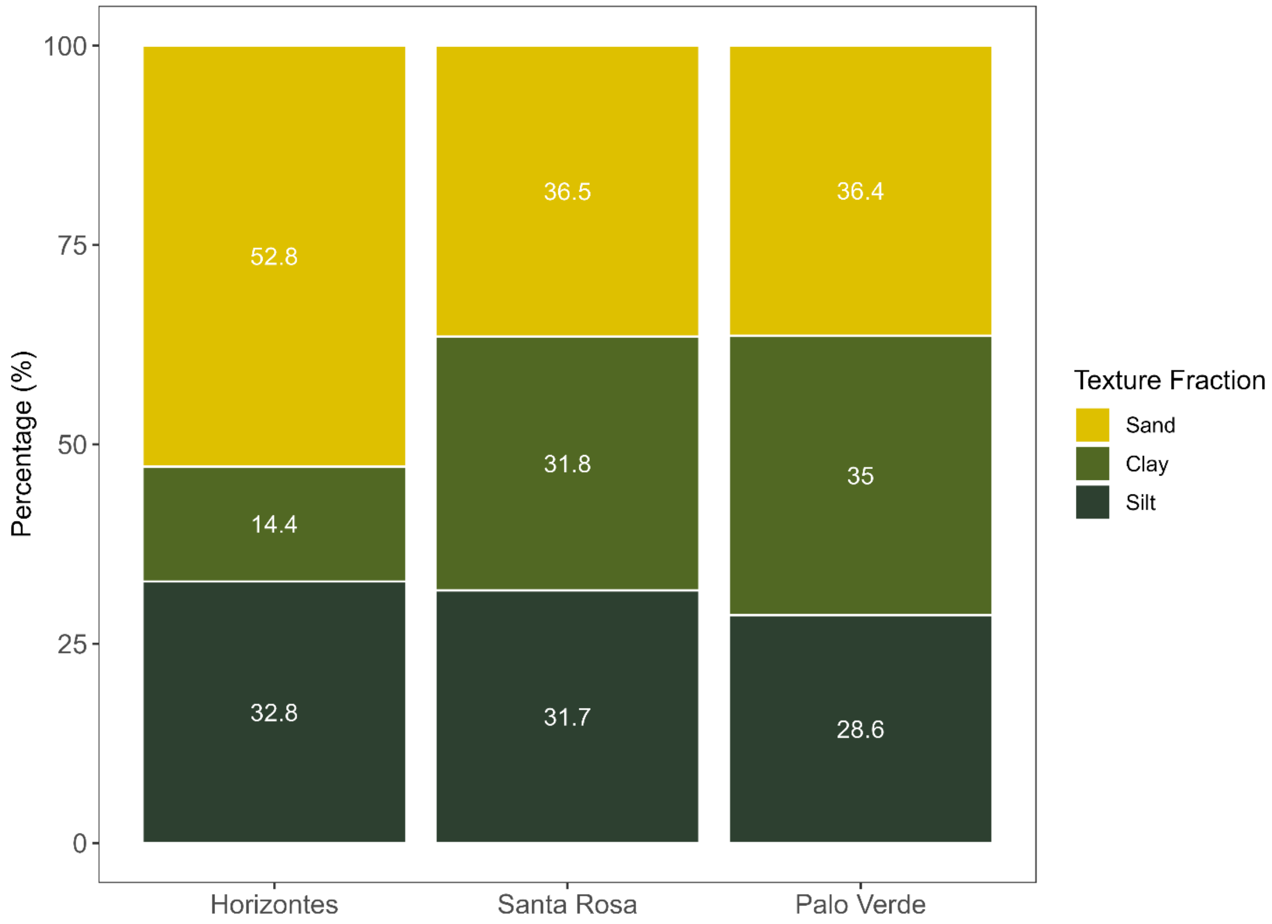

Figure S2. Mean soil texture fractions for each site in Guanacaste, Costa Rica (N = 15).

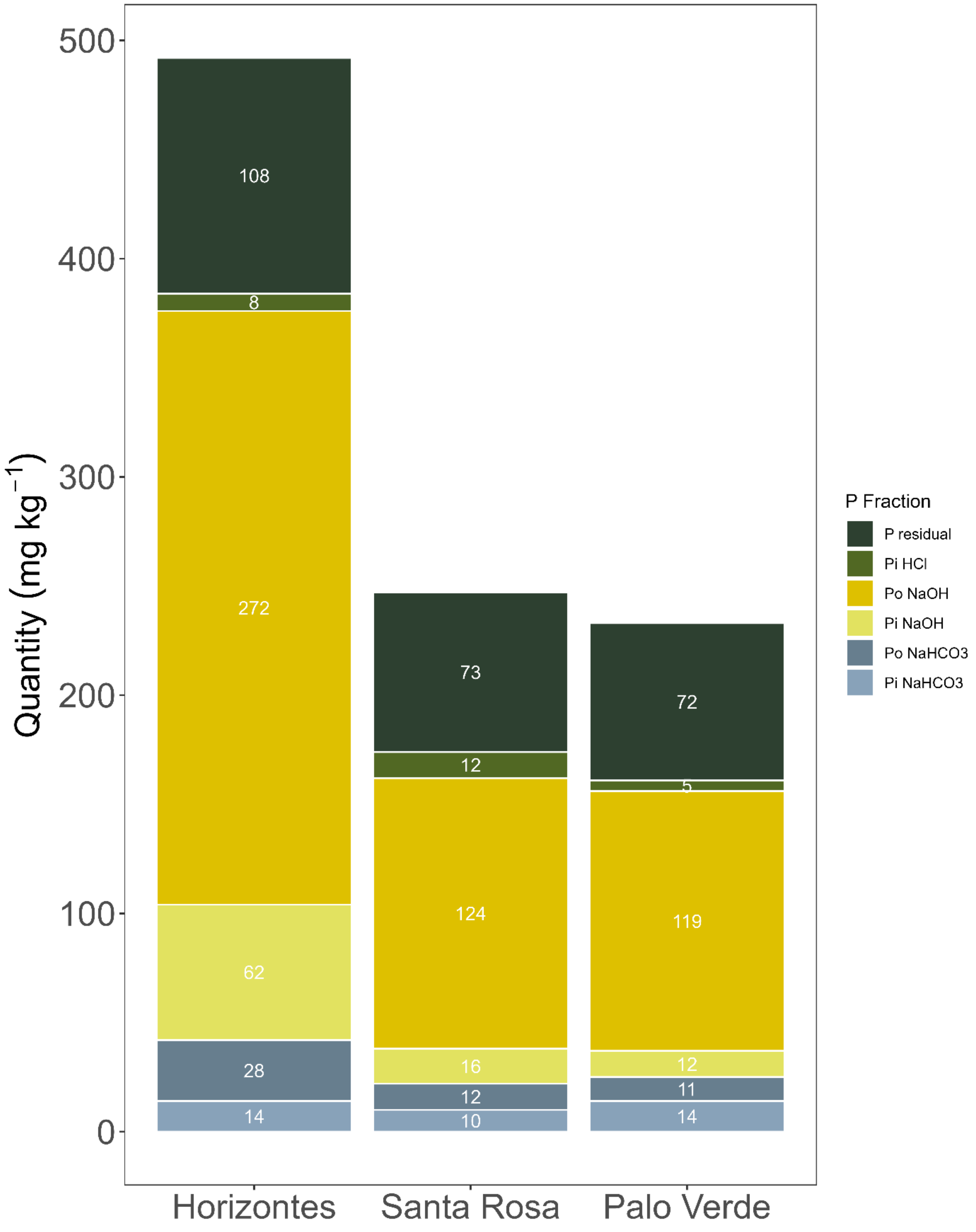

Figure S3. Mean Hedley phosphorus (P) fractions for soil samples pooled between the two nearest trees in each site (N = 15) in Guanacaste, Costa Rica. Pi = inorganic P, Po = organic P
